# Suicidal ideation and its related factors among older adults: a population-based study in Southwestern Iran

**DOI:** 10.1186/s12877-022-03049-9

**Published:** 2022-04-28

**Authors:** Ramin Shiraly, Hamideh Mahdaviazad, Roya Zohrabi, Mark D. Griffiths

**Affiliations:** 1 Department of Community Medicine, School of Medicine, Shiraz University of Medical Sciences, Shiraz, Iran; 2 Health Behavior Science Research Center, Shiraz University of Medical Sciences, Shiraz, Iran; 3 Student Research Committee, Shiraz University of Medical Sciences, Shiraz, Iran; 4 International Gaming Research Unit, Psychology Department, Nottingham Trent University, 50 Shakespeare Street, Nottingham, NG1 4FQ UK

**Keywords:** Older adults, Suicidal ideation, COVID-19, Pandemic, Depression, Social support

## Abstract

**Objectives:**

Suicidal ideation is a major risk factor for suicide and can negatively affect self-care and health behaviors among the older adults. There are limited data on the prevalence and risk factors of suicidal ideation among the older population during the COVID-19 pandemic. The aim of the present study was to examine the prevalence and risk factors of suicidal ideations among Iranian older adults during the COVID-19 pandemic.

**Methods:**

A total of 803 older community adults in Shiraz (Southwestern Iran) were surveyed to determine potential factors influencing suicidal ideation, including demographic factors, physical health status, access to healthcare, current depression status, fear of COVID-19, perceived social support, and social engagement. Data were collected utilizing face-to-face interviews between November and December 2020. Multivariate logistic regression analysis was used to identify independent variables associated with suicidal ideations.

**Results:**

Among the 803 participants, 69 reported suicidal ideations (8.6%). Individuals with suicidal ideations were more likely to have greater fear of COVID-19. However, based on the results of multivariate logistic regression analysis, current depression (OR: 2.07, CI 95%: 1.18–3.65), not being married (OR: 1.82, CI 95%: 1.06–3.13), inability to pay for medical bills (OR: 2.16, CI 95%: 1.23–3.79), low perceived social support (OR: 2.03, CI95%: 1.11–3.71), and having limited social network (OR:1.77, CI 95%: 1.02–3.10) appeared to be more powerful influencing factors.

**Conclusion:**

Suicidal ideation appears to be relatively common among Iranian older adults during the COVID-19 pandemic. A lack of longitudinal data makes it difficult to establish an association between suicidal ideations and the COVID-19 pandemic. Systematic monitoring of suicidal ideation is recommended among high-risk groups, particularly the older population.

## Introduction

Late life suicide remains a major global health problem, with those individuals aged 65 years and over constituting the demographic group with the highest suicide rate in most countries according to the World Health Organization [1, 2]. In addition to being a major risk factor for suicide [3, 4] suicidal ideation (SI) can lead to a number of negative consequences in older adults such as poor self-care and increased mortality irrespective of baseline depressive status [5]. Estimates of SI prevalence among older adults vary widely across the world and ranges from 3 to 25% [6–9]. Studies show that prevalence of SI is particularly high among older adults who are single, widowed, bereaved, living alone and/or being socially isolated, as well as having underlying physical and/or mental disorders [10]. There are no reports concerning the prognosis of SI among older adults. However, for individuals of different ages who report SI within the previous 12 months, one-year prevalence rates of suicidal acts have been estimated to be 15–20% [11].

Suicidal behavior (SB) has been described as deliberate injury to self with the purpose of ending one’s life. Suicidality exists along a spectrum of severity with different levels of suicidal thoughts representing different levels of suicide risk. It can range from passive desire for death, to active thoughts of killing oneself, and in the most extreme cases having a specified plan with the intention to die by suicide [11, 12] SI is a significant risk factor for future suicide and is associated with higher risk of suicidal attempt (SA) and death [13]. The global lifetime prevalence for suicidal thoughts and suicide attempts among all age groups combined have been estimated to be 9 and 3%, respectively [3].

The coronavirus disease 2019 (COVID-19) pandemic is another potential factor that might be a contributor to SI. Pandemics can be stressful and increase anxiety levels among healthy individuals and escalate the symptoms of individuals with pre-existing mental health conditions. Pandemics can trigger a wide range of psychosocial impacts such as extreme anxiety, anger, insomnia, social isolation, depression, somatization, and increased use of alcohol and tobacco [14, 15]. Moreover, older adults are also at an elevated risk of late life depression and suicide. Depressive symptoms among older adults may be accentuated by stressful events [16].

Another psychological consequence of pandemics is fear. Fear is an adaptive response to potentially threatening situations. However, it can contribute to development of psychiatric disorders when it is excessive or disproportionate to real risks [17]. Fear can facilitate coping with unfavorable or unanticipated situations. However, pathological anxiety can impede an individual’s ability to cope effectively with life challenges and crises and can lead to functional impairment and chronic psychiatric disorders. Susceptibility to pathological fear and anxiety appears to be the result of predisposing factors and traits [18, 19].

As an international health crisis, the COVID-19 pandemic has affected millions of individuals all around the world. It has evoked much public fear and anxiety and the psychological impacts of the crisis have been extensive [20]. Across different age groups, the geriatric population is particularly vulnerable to this viral respiratory infection. Older individuals account for the majority of COVID-related deaths and are at higher risk of developing severe symptoms. As a cohort, they are also more concerned about being infected and are more inclined to stay at home during pandemics [21].

There is limited evidence on the effect of the COVID-19 pandemic on mental health and SB of older adults at the population level. However, to date, most published findings imply that older adults may be less negatively affected by mental health outcomes of the pandemic than other age groups [22]. According to a study conducted examining more than 5000 people in the United States, older adults reported lower rates of SI in the preceding 30 days than other age groups [23]. A population-based study conducted during the initial phase of COVID-19 pandemic in Spain also showed that being older (aged 60 to 80 years) was negatively associated with symptoms of both depression and anxiety [24]. Another study that assessed loneliness and mental health status among Dutch older adults during early phase of the pandemic showed that although social and emotional loneliness increased, mental health issues remained unchanged during the pandemic compared to pre-pandemic months [25].

Analysis of suicide statistics from several countries showed that during the pandemic, suicide death rates initially declined or remained unchanged [26]. However, in the case of Japan, after an initial decline there was an increase in suicide deaths, particularly among young female workers who had experienced job loss [27]. A longitudinal study across the pandemic waves in UK reported an increased rate of suicidal thoughts, especially among young female adults and those with preexisting mental health problems [28]. There have also been case reports of COVID-related suicides among older adults with a history of preexisting mental illnesses [29].

The current pandemic period is a unique timeframe in human history when an international health crisis has affected physical and mental well-being of millions of individuals globally. This unprecedented situation can be considered as an influential social phenomenon that may interact with psychological variables to increase vulnerability or resilience to mental illnesses [16]. However, it has been shown that SI can be influenced by various social and cultural factors [2]. Therefore, the aim of the present study was to examine the prevalence and risk factors of SI among a sample of Iranian older adults in Shiraz, Southwestern Iran during the COVID-19 pandemic.

## Methods

### Study setting and participants

The present study was conducted in Shiraz, a city located in the Fras Province in Southwestern Iran which has a population of approximately 2 million inhabitants of which approximately 160,000 are aged over 60 years. Participants were recruited utilizing a household survey conducted through face-to-face interviews within urban neighbourhoods of the city using a multi-stage cluster sampling. The inclusion criteria were being Iranian, aged 60 years and older, and being able to understand and respond to the survey questions. All participants said they were cognitively capable of participating in an interview in Persian and no-one was excluded from participating in the study in relation to this criterion. Participation was voluntary and no financial remuneration was provided. The sampling approach was based on clusters from municipal areas, then neighborhoods. In the first stage, municipal areas were numbered and six (out of 11) regions were randomly selected. In the next stage, a total of 17 neighborhoods were randomly selected from municipal regions proportional to their population size. In the final stage, 50 participants were randomly selected from each neighborhood. Within the neighborhoods, a list of households who had eligible members according to age was prepared and a simple random selection procedure was applied. For each selected household, if there was more than one eligible person, one of them was randomly selected. A total of 850 older adults were invited to be interviewed and 803 participated in the study (response rate: 94.5%). The mean age of participants was 68.1 years (SD = 4.73). Participants provided verbal consent to participate prior to commencing the study. Data were collected from the 24 November to 20 December 2020, during the COVID-19 pandemic in Iran. The survey took approximately 45 min for every participant to complete. Interviews were conducted outdoors while maintaining adequate physical distance (at least two meters) and wearing face masks. The protocol was approved by the Shiraz University’s Ethics Committee (Ref: IR.sums.med.rec.1399.518). Written informed consent was obtained from all participants, after they had been informed of the study’s goals. Complete anonymity and data confidentiality was guaranteed. The research conducted in this study was performed in accordance with the Declaration of Helsinki.

### Measures

#### Demographics

Basic demographic data included age, gender, marital status, working status and educational level were collected.

#### Access to healthcare

Self-reported access to healthcare was assessed using three questions. Questions included whether participants had (i) medical insurance coverage, (ii) easy access to a physician, (iii) problems in paying for medical bills during past 12 months.

#### Physical health status

Self-reported physical health status was assessed using a single question: (“How would you generally rate your current physical health?”) rated on scale from 1 (*very poor*) to 5 (*excellent*). Participants were also asked if they had any of the following common chronic medical conditions that necessitated regular medical visits and medication use: hypertension, heart disease, stroke, cancer, chronic lung disease and/or chronic kidney disease.

#### Current depression

The Persian version of the two-item Patient Health Questionnaire-2 (PHQ-2) was used to assess the presence of depression among participants [30]. The questions ask participants if they had “Little interest or pleasure in doing things” and/or were “Feeling down, depressed or hopeless” during the past 2 weeks. The responses are rated from 0 to 3 (0 = not at all, 1 = several days, 2 = more than half the days, and 3 = nearly every day) with the total score ranging from 0 to 6. A score of 3 or more is 83% sensitive and 90% specific for a diagnosis of major depression. Both the PHQ-9 and PHQ-2 are reliable and valid tools for screening and assessment of depressive symptoms [31, 32]. Cronbach’s alpha in the present study was 0.838.

#### Suicidal ideation and behaviors

To evaluate the presence of SI, three questions adapted from Ask Suicide-Screening Questionnaire (ASQ) [33] were used. This five-item scale assesses recent SI (first three questions) and lifetime and present SB (final two questions). The ASQ items are responded to either ‘yes’ or ‘no’ and comprise: “1=In the past few weeks, have you wished you were dead?”, “2=In the past few weeks, have you felt that you or your family would be better off if you were dead?”, “3=In the past week, have you been having thoughts about killing yourself?”, “4=Have you ever tried to kill yourself?” and “5=Are you having thoughts of killing yourself right now?”, with the final question assessing severity [27]. A ‘yes’ answer to the any of the first three questions were considered as recent suicidal ideation state. Previous studies have shown that the ASQ tool has robust psychometric properties among a wide range of ages. For example, in a sample of 727 adults (mean age: 50 years, range: 18–93 years), the ASQ showed a high sensitivity and specificity for screening of SI (100 and 89%, respectively) [34]. Following the cross-cultural scale translation and validation guidelines by Sousa and Rojjanasrirat (2011) [35], the items were translated into Persian and backward translated to English by two independent Persian-speaking individuals who were proficient in English. Discrepancies between the original questions and the back-translated version were discussed and resolved by the research team. Face validity and content validity were assessed by five experts including one linguist and construct validity was evaluated by factor analysis. The internal consistency was measured utilizing Cronbach’s alpha. The means for the content validity index and the content validity ratio were 0.89 and 0.85, respectively. Cronbach’s alpha value was 0.762. The translated Persian version of the questions were therefore internally consistent, reliable, and valid. The strong psychometric properties in relation to validity and reliability were consistent with previous studies [33, 34, 36].

#### Fear of COVID-19

The seven-item Fear of COVID-19 Scale (FCV-19S) [37] originally validated using an Iranian sample was used to assess fear of COVID-19. Items (e.g., *“I am afraid of losing my life because of COVID-19”*) are rated on a five-point scale from 1 (*strongly disagree*) to 5 (*strongly agree*) with total scores ranging from 7 to 35. Higher scores denote greater COVID-19-related fear. The FCV-19S was used because it has been validated in many languages with good psychometric properties [38]. Cronbach’s alpha in the present study was 0.918.

#### Social engagement

The Persian version of the six-item Lubben Social Network Scale (LSNS-6) [39] was used to assess social engagement among family and friends. Items (e.g.*, “How many relatives do you see or hear from at least once a month?”*) are rated on a five-point scale from 0 (*none*) to 5 (*nine or more*) with total scores ranging from 0 to 30. A lower score indicates an increased risk for social isolation [38, 40]. Cronbach’s alpha in the present study was 0.871.

#### Perceived social support

The Persian version of 12-item Multidimensional Scale of Perceived Social Support (MSPSS) [41] was used to assess perceived social support from three different sources (family, friends and a significant other) on three subscales Items (e.g., *“I get the emotional help and support I need from my family”*) are rated on a seven-point scale ranging from 1 (*very strongly disagree*) to 7 (*very strongly agree*) with total scores ranging from 12 and 84. A higher score indicates greater social support perceived by an individual. Cronbach’s alpha in the present study was 0.914.

### Statistical analysis

Descriptive statistics were used to calculate the main sample characteristics of the study participants. Demographic factors were calculated as frequencies and percentages for categorical variables, and means and standard deviations for numerical variables. Chi-square tests and the independent sample *t-*tests were used to calculate statistical differences between those who reported and those who did not report SI. To assess effect of social engagement, and perceived social support on the risk of SI, LSNS-6 and MSPSS scores were dichotomized into two categories using the median value as a cutoff point. Multivariate logistic regression was utilized to determine independent variables associated with increased risk of SI. When building the multivariate models, each of the variables listed above were evaluated in univariate models, and those with *p*-values <.10 were considered for inclusion in the backwards stepwise logistic regression. SPSS version 28 (IBM, United States) was used for all statistical analysis and significance level was set at *p* < .05.

## Results

Of the 803 participants, 69 reported SI in the past few weeks (8.6%), 12 had thoughts of killing themselves at the time of interview (1.5%), and 16 had previously attempted suicide at least one during their lives (2%). Table 1 shows the characteristics of older adults who did and did not report SI. Those reporting SI were significantly more likely to (i) be female (61% vs. 48%), (ii) be unmarried (42% vs. 24%), (iii) be unable to pay medical bills in the past 12 months (38% vs. 18%), (iv) have poor to moderate physical health (71% vs. 53%), (v) have a history of chronic disease (55% vs. 35%), (vi) be experiencing current depression (35% vs. 16%), (vii) have greater fear of COVID-19, (viii) have low social engagement (70% vs. 53%), and (ix) have low perceived social support (77% vs. 58%).

**Table 1:** Characteristics of study participants

Characteristics	Without suicidal ideation (*n* = 734) N (%)	With suicidal ideation (*n* = 69) N (%)
**Demographics**		
Age, mean (SD)	67.8 ± 4.7	68.1 ± 4.8
Gender, n (%)		
*Male*	381 (51.9)	27 (39.1)^*^
*Female*	353 (48.1)	42 (60.9)
Marital status		
*Married*	556 (75.7)	40 (58)^**^
*Single/divorced/widowed*	178 (24.3)	29 (42)
Work status, n (%)		
*Working*	227 (30.9)	16 (23.2)
*Retired/housewife*	507 (69.1)	53 (76.8)
Educational level, n (%)		
*Illiterate/primary school*	474 (64.6)	52 (75.4)
*High school/college*	260 (35.4)	17 (24.6)
**Access to healthcare**		
Health insurance, n (%)		
*No*	81 (11)	12 (17.4)
*Yes*	653 (89)	57 (82.6)
Easy access to medical care, n (%)		
*No*	59 (8)	8 (11.6)
*Yes*	675 (92)	61 (88.4)
Unable to pay medical bills in the past 12 months		
*Yes*	134 (18.3)	26 (37.7)^***^
*No*	600 (81.7)	43 (62.3)
**Physical health status**		
Self-rated physical health, n (%)		
*Poor to moderate*	390 (53.1)	49 (71)^**^
*Good to excellent*	344 (46.9)	20 (29)
History of chronic disease, n (%)		
*No*	480 (65.4)	31 (44.9)^**^
*Yes*	254 (34.6)	38 (55.1)
**Mental health status**		
Current depression by PHQ2		
*No*	616 (83.9)	45 (65.2)^***^
*Yes*	118 (16.1)	24 (34.8)
Fear of COVID-19, mean (SD)	20.66 ± 6.4	22.20 ± 6.9^*^
**Social engagement**		
Social networks by LSNS-6		
*Limited (<=12)*	386 (52.6)	48 (69.6)^**^
*Adequate (> 12)*	348 (47.4)	21 (30.4)
Perceived social support by MSPSS		
*Low (<=60)*	425 (57.9)	53 (76.8)^**^
*High (> 60)*	309 (42.1)	16 (23.2)

*LSNS* Lubben Social Network Scale, *MSPSS* Multidimensional Scale of Perceived Social Support, *FCV-19S* Fear of COVID-19 Scale

^*^p < .05

^**^p < .01

^***^p < .001

Table 2 shows the association between predisposing factors and the risk of SI. Independent factors for SI among Iranian older adults were current depression (OR: 2.07, CI 95%: 1.18–3.65), being unmarried (OR: 1.82, CI 95%: 1.06–3.13), having financial problems in paying for medical bills (OR: 2.16, CI 95%: 1.23–3.79), having low perceived social support (OR: 2.03, CI 95%: 1.11–3.71), and having a limited social network (OR:1.77, CI 95%: 1.02–3.10).

**Table 2:** Factors associated with suicidal ideation

Factors	Univariate model		Multivariate model	
	OR (95% CI)	*p*-value	OR (95% CI)	*p*-value
Age in years	0.98 (0.94–1.04)	0.604	–	–
Female sex	1.68 (1.01–2.78)	0.044	1.31 (0.76–2.24)	0.327
Single/widowed/divorced status	2.26 (1.36–3.76)	0.002	1.82 (1.06–3.313)	0.030^*^
Not working	1.48 (0.83–2.65)	0.183	–	–
Low educational level	1.67 (0.95–2.96)	0.074	1.15(0.62–2.13)	0.664
No insurance	1.69 (0.87–3.29)	0.118	–	–
Difficulty in access to medical care	1.50 (0.68–3.28)	0.310	–	–
Inability to pay medical bills in the past 12 months	2.70 (1.60–3.28)	< 0.001	2.16 (1.23–3.79)	0.008^*^
Poor to moderate physical health	2.16 (1.25–3.70)	0.005	1.43 (0.78–2.61)	0.245
History of chronic disease	2.31 (1.40–3.81)	0.001	1.61 (0.95–2.74)	0.078
History of diagnosed depression/anxiety	3.47 (1.98–6.10)	< 0.001	1.77 (0.91–3.44)	0.091
Current depression	2.78 (1.63–4.74)	< 0.001	2.07 (1.18–3.65)	0.011^*^
Fear of COVID-19	0.96 (0.92–1.01)	0.057	1.60 (0.90–2.82)	0.104
Limited social network	2.06 (1.20–3.51)	0.008	1.77 (1.02–3.08)	0.042^*^
Low perceived social support	2.40 (1.35–4.29)	0.003	2.03 (1.11–3.71)	0.021^*^

^*^Statistically significant

## Discussion

The present study examined the prevalence and factors associated with SI among older adults in an urban Shiraz community. To our best of the present authors’ knowledge, this is the first study in Iran to assess SI among older people. The prevalence of suicidal thoughts among the studied population during the COVID-19 pandemic was 8.6%. There is a dearth of population-based studies assessing SI among Iranian older adults. Pre-pandemic studies from other countries show a diverse range of prevalence rates. The prevalence of SI have been reported to be 23% among older individuals of rural Bangladesh [42], 19.6% among Korean older adults [43], and 16.5% among community dwelling Taiwanese older people [44]. In a cross-sectional population-based survey in Mexico, the lifetime prevalence of SI among individuals aged 65 years and over was 13.5 and 4.5% in the past 2 weeks [45]. Two studies from Western countries (United States and the United Kingdom) reported prevalence rates of ever having had suicidal thoughts among older adults to be 6 to 7% [46]. Also, a Swedish study reported a prevalence rate of 16% among individuals 80 years and older [46]. However, it should be noted that these different studies have used different tools which that makes direct comparison difficult.

Considering SI is a pivotal component of the suicidal process, global differences in suicidal behaviors can also be observed in suicide death rates across the world. Globally, there is a considerable inter-country variability in suicide rates [11]. Whole population suicide rates range from approximately 20 per 100,000 across Eastern Europe, South Korea, Zimbabwe, Guyana, and Suriname to less than five per 100,000 across North Africa, the Middle East, Indonesia, Peru, and some Mediterranean countries [47]. According to WHO reports, the Eastern Mediterranean region has the lowest age-standardized suicide rates (four per 100,000 population, 2016) among world WHO regions [48]. This has been attributed to cultural, religious, and social factors in this region [49]. Cultural diversity may account for a part of significant variations in suicide rates across the world. It is also assumed that family support is a strong predictor of maintaining psychosocial health and mental well-being [50]. Social support prevents the negative mental outcomes of stressful life events and a high level of social support decreases the risk of suicide [51]. The findings of the present study showed low perceived social support as an independent risk factor for SI among Iranian older adults.

Based on the findings of the present study, inability to pay for medical bills in the past 12 months was another independent variable influencing SI among Iranian older adults. Although the present study considered this as a measure of access to healthcare, it may also reflect the economic status of the study participants. It is well known that low economic status is a risk factor for suicide among the general population [11].

While the findings of the present study showed that SI was more common among female than male older adults, gender was not identified as an independent factor associated with SI. Most studies on gender differences of SI have compared suicide death rates and SA. Death rates from suicides are four to five times higher for males than female, and females demonstrate a disproportionately higher rate of SA compared to males [52]. Although reports on gender difference in SI are less explicit, a majority show a female preponderance [8, 53].

The findings of the present study showed that current depression was a significant risk factor for SI in the target population. SI among older adults is mostly seen in the context of mental disorders. In a US study of the older people aged 65 years and over in primary care settings, almost half of those with depression or anxiety reported SI [54]. In a cross-sectional study across 17 countries, the presence of any anxiety disorder increased the risk of SI threefold and mood disorder increased the risk fivefold [3].

Historically, international crises have been associated with increase in suicide rates. Rises in national suicide rates following the 2008 global economic crisis were particularly seen among young and middle-aged men and appeared to be associated with a significant increase in unemployment [55]. It is expected that under socially stressful conditions like a fatal pandemic disease, older individuals are more likely to feel scared and be worried about possible consequences and perceive social problems as impossible to resolve and therefore they might experience increased SI and SB [56]. An increase in SA among older individuals was reported after the severe acute respiratory syndrome (SARS) epidemic in Hong Kong in 2003–2004 [57]. Although participants in the present study who reported SI were more likely to report fear of COVID-19 pandemic, this variable was not a robust independent influencing factor based on multivariable regression analysis. Given the study’s limitations, this finding should be treated cautiously. One interpretation is that fear of COVID-19 mostly affects people with pre-existing mental health conditions, such as depression. Another explanation refers to Iranian cultural context. Traditionally, Iranian families strongly provide support to family members [58]. Family cohesion and support has also been suggested as a protective factor in coping with psychological distress in some other cultures such as Latino families [59]. However, caution is warranted given the cross-sectional design of the present study.

In summary, the present study estimates that 1 in 12 Iranian older adults experienced SI during the COVID-19 pandemic. After conducting multivariable logistic regression analysis, the main influencing factors were interconnected and mostly related to social and economic domains. Based on these findings, it is recommended that Iranian health policymakers should focus on strategies to identify and address factors contributing to the socio-economic inequalities in access to healthcare and social support services among vulnerable groups, particularly the older population. Strategies should therefore be directed at improving the usability and availability of social facilities, activities, and resources for underserved older adults, all of which are generally associated with improved mental health outcomes [15, 60, 61]. Practical examples of such strategies might include facilitating access to healthcare by providing older adult-specific health insurance, ensuring healthcare provider availability and continuity, home visiting programs, facilitating access to public spaces by providing older adult-friendly transportation services and enhancing community safety (particularly for those with functional disabilities), peer support programs, and providing information and communication technologies such as applications and social media platforms to promote social engagement. Moreover, such strategies must be customized to address the socio-cultural needs and preferences of the older adults at both country and sub-country levels. Given the commonality of SI among Iranian older adults, investigating its presence in primary care practice settings is warranted.

There are some limitations that should be noted. The present study was conducted utilizing a modest sample size of individuals in a province of Iran, so the study findings may be not generalizable to all Iranian older adults. A further limitation is the aforementioned cross-sectional design of the study. The present study only reported risk factors associated with SI among Iranian older adults during the pandemic and cannot say anything about causation. Longitudinal studies are needed to confirm the associations found between variables in the present study. However, despite its limitations, the data provided an estimate of the prevalence of SI in the target population and provided some preliminary inferences in the absence of longitudinal data. Insomnia has also been suggested as a risk factor for late life depression and suicide [62]. However, the present study did not assess this variable among the studied population. The single item self-reported physical health status has been shown to be predictive of future morbidity and mortality [63]. However, it does not focus on specific health aspects during a specific timeframe, but rather provides individuals’ own judgments of their perceived general health. Moreover, all of the data were self-report which are subject to a number of well-known methods biases. This should also be taken into account when interpreting the study’s main findings.

## Conclusion

SI are relatively common among Iranian older adults during COVID-19 pandemic. A lack of longitudinal data makes it difficult to establish an association between SI and the COVID-19 pandemic. Implications for health policy include developing and implementing strategies to increase social connectedness, particularly during the pandemic setting, and to improve social support for Iranian underserved older adults. Also we recommend systematic monitoring of SI among the older population. This policy can be integrated into existing comprehensive geriatric assessment in primary health care.

## Data Availability

The datasets used and/or analyzed during the current study are available from the corresponding author on reasonable request.

## Abbreviations

PHQ-2: Patient Health Questionnaire-2
ASQ: Ask Suicide-Screening Questionnaire
FCV-19: Fear of COVID-19
LSNS: Lubben Social Network Scale
MSPSS: Multidimensional Scale of Perceived Social Support
SARS: Severe Acute Respiratory Syndrome
